# Benchmarking heterogeneous network-based methods for drug repurposing

**DOI:** 10.1038/s41540-025-00633-8

**Published:** 2025-12-10

**Authors:** Thi Trang Nguyen, Yudi Pawitan, Stefano Calza, Trung Nghia Vu

**Affiliations:** 1 Department of Medical Epidemiology and Biostatistics, Karolinska Institutet, Stockholm, Sweden; 2 Department of Molecular and Translational Medicine, University of Brescia, Brescia, Italy

**Keywords:** Biochemistry, Computational biology and bioinformatics, Drug discovery

## Abstract

Drug repurposing (DR) has gained significant attention as a cost-effective strategy for identifying new therapeutic uses for existing drugs. Heterogeneous network-based methods are particularly promising because they exploit complex biological interactions. However, comprehensive benchmarking across multiple datasets is still needed to assess their reliability and generalizability. We systematically evaluate ten advanced heterogeneous network-based DR methods across eight datasets, including six publicly available and two newly introduced drug-disease datasets. The methods include (i) matrix factorization: NMF, NMF-PDR, NMF-DR, VDA-GKSBMF, (ii) matrix completion: BNNR, OMC, HGIMC, (iii) recommendation systems: IBCF, LIBMF, and (iv) a deep learning approach: DRDM. Performance is assessed using the area under the receiver operating characteristic (AUC) and precision-recall curve (AUPR). We also analyze the impact of data sparsity and compare findings with previous benchmarking studies. Our results reveal that OMC consistently achieves the highest AUC and AUPR across most datasets. BNNR, DRDM, HGIMC, VDA-GKSBMF, and NMF-PDR, also demonstrate competitive performance, with NMF-PDR outperforming other NMF-based approaches. We find that differences in cross-validation strategies substantially impact reported AUPR values, with previous studies overestimating performance by omitting many negative instances. This work provides a reliable benchmarking framework and new datasets, offering insights for future research in DR.

## Introduction

Drug repurposing (DR), also known as drug repositioning, refers to the process of identifying new therapeutic uses for existing drugs beyond their original use or approved indication^1^. DR provides significant benefits over traditional drug discovery methods, including lower timeline, cost-effectiveness, and reduced risk^2^. Recently, computational DR has become highly promising due to its numerous advantages, such as ability to perform large-scale screening, and effective utilization of existing data.

Among computational approaches, heterogeneous network-based methods, which predict drug-disease association by integrating information from drug networks, disease networks and a drug-disease interaction networks, have demonstrated outperformance against other computational DR methods^3–8^. The principle for the approaches is that drugs with similar properties tend to target related diseases^9^.

In the category of machine learning (ML) approaches, kernel-based methods are effective at integrating heterogeneous similarity measures within a regularized least squares (RLS) framework. Early work, such as KronRLS^10^ applied RLS to the Kronecker product of drug and target similarity kernels, while extensions, such as KronRLSWNN^11^ addresses unseen drugs, and KronRLS-MKL^12^ improves kernel selection by multiple kernel learning. More recently, pairwiseMKL^13^ enhances scalability by jointly optimizing kernel weights and prediction functions in a memory and time efficient manner for large-scale bioactivity prediction.

Finally, graph neural networks (GNNs), a deep learning technique, has been increasingly used for DR because it can capture complex patterns from high dimensional biomedical data^14–17^. For example, TGCNDR^18^ integrates drug, protein, disease, and side-effect data in a tripartite cross-network with attention-based message passing, enabling more comprehensive learning of biological associations. Additionally, contrastive learning has been applied to improve embeddings by keeping them consistent across multiple network views^19,20^. For example, AutoDR^21^ enhances LightGCN with neighborhood recalibration and dual contrastive losses to strengthen collaborative signals and reduce feature redundancy, while DRDM^22^ applies an adaptive debiasing mechanism within graph message passing to mitigate popularity bias and incorporates dual-view contrastive learning to improve generalization.

Heterogeneous network-based DR methods can be categorized into several main groups: ML, network propagation, matrix factorization (MF), and matrix completion (MC) -based approaches. A recent evaluation of 28 heterogeneous network-based DR techniques^8^ suggested that the MC and MF methods demonstrated the best performance. Further benchmarking and reviews from diverse perspectives have been performed for a comprehensive understanding of DR methodologies^3,4,6,7,23,24^.

However, existing evaluations are often subject to certain limitations. For example, many studies^24–28^ rely solely on Area Under the Curve (AUC) as the primary comparison metric, and neglects the Area Under the Precision-Recall Curve (AUPR) in assessment. While AUC is widely used, it can be misleading in highly imbalanced datasets like drug-disease association matrices (typically >90% negative associations). AUPR is more appropriate for the sparse matrices, as it focuses specifically on the performance of the model on positive cases (true drug-disease associations).

Furthermore, several evaluation studies^8,29,30^ report unrealistically high AUPR (>0.90) for DR methods, raising concerns about validation methodologies. For example, one approach^8^ performs cross-validation only on positive associations, but report results on balanced test sets (where negative samples are randomly subsampled). Thus, the majority of the negative associations are excluded from the evaluation, which might inflate performance. Another concern is information leakage in validation, where the same information is inadvertently used in both training and validation sets. Finally, most benchmarking studies lack detailed descriptions of validation procedures and publicly available source codes, making it difficult to reproduce results and reconcile conflicting conclusions across studies^8^.

To address these limitations, we propose a comprehensive benchmarking framework for assessment of computational DR methods. This framework will employ both AUC and AUPR as the comparison metrics and implement a suitable evaluation approach based on a disease-centric cross-validation, aiming fair and reproducible comparisons. This study focuses on evaluating MC and MF-based methods, including (i) five top performing models from a recent benchmarking study^8^: HGIMC^4^, BNNR^31^, VDA-GKSBMF^32^, OMC^33^, and NMF-DR^27^; (ii) standard NMF^34^; (iii) NMF-PDR, a novel permutation approach for NMF in DR; (iv) two methods including IBCF^35^ and LIBMF^36,37^ which are widely used in recommendation system (RS) but not well investigated in previous DR benchmarking studies; and (v) a recently deep learning method DRDM^22^, which shows better performance than GCN-based models (DRHGCN) in a recent benchmarking study^8^. We evaluate these DR methodologies using six public DR datasets and two newly constructed datasets: one dataset includes all diseases, while the other focuses on rare diseases. The new datasets are built using MechDB, the largest curated drug-disease database^38^, incorporating disease symptoms to define disease-disease similarity.

## Results

### Overall performance of DR methods across datasets

**Fig. 1:**
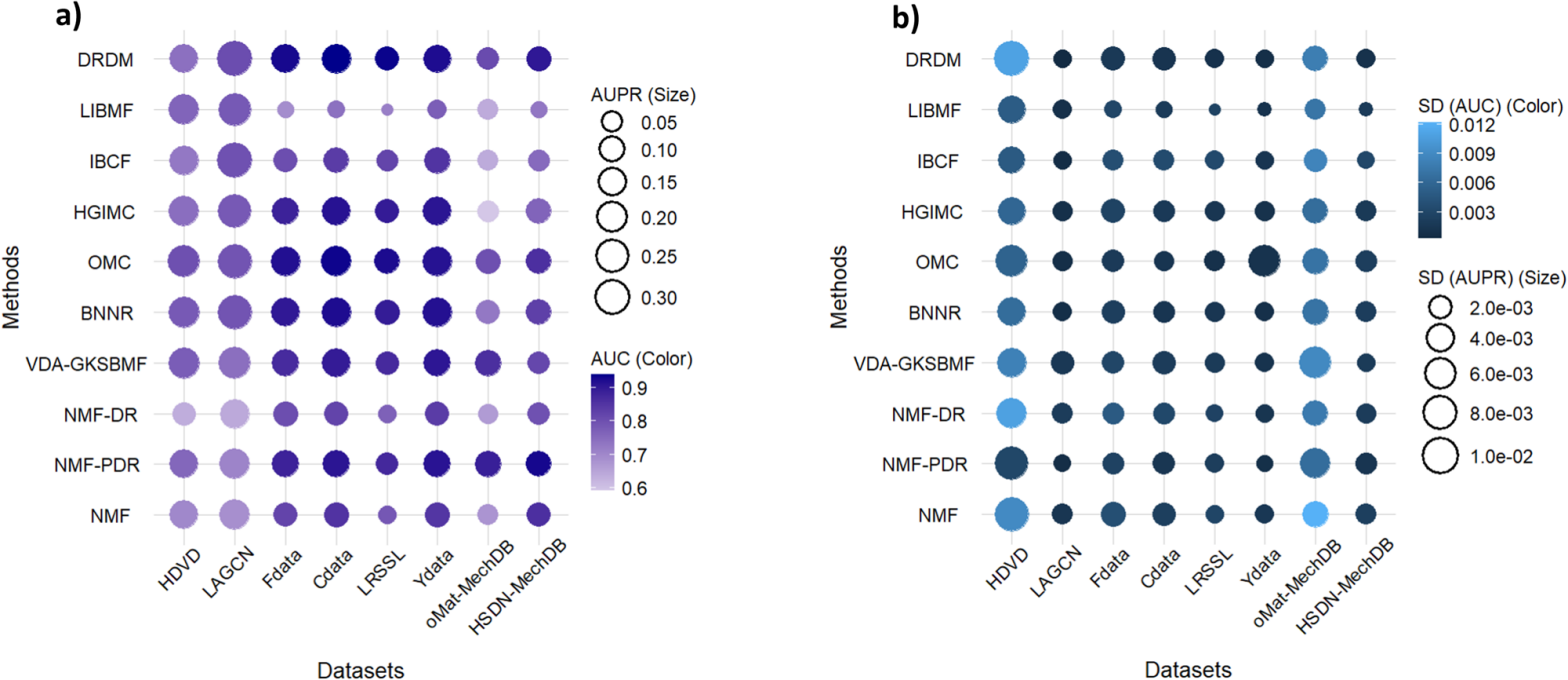
The model performance for 10-fold cross-validation across 25 runs for each method and dataset. **a** Heatmap of median AUC values. **b** Heatmap of median AUPR values. In each square, the color represents the AUC (AUPR) median value, the number inside indicates the exact AUC (AUPR) median value, and the color of the number denotes the corresponding standard deviation (SD).

Overall results are shown in Fig. 1, presenting the median AUC (Fig. 1a) and median AUPR (Fig. 1b) values across 25 CV runs. In these heatmaps, each square represents a method (row) evaluated on a dataset (column), where the color intensity denotes the median AUC or AUPR value, and the color of the number inside each square indicates the corresponding standard deviation (SD). These results indicate that no single method consistently outperforms all others across all datasets. The results of all methods are highly stable with SD <0.01 for both AUPR and AUC across 25 runs. The details of the AUC and AUPR values are provided in Supplementary Tables S1 and S2.

In general, OMC, BNNR, and DRDM are generally among the top performing methods in most datasets for both AUC and AUPR, followed by HGIMC, VDA-GKSBMF, and NMF-PDR, as shown in the ROC and Precision-Recall (PR) curves in Supplementary Figs. S1 and S2, respectively. For datasets, LAGCN obtains the highest AUPR while Fdata, Cdata and Ydata achieve the highest AUC across methods. The further details are described in next sections.

### Method-specific performance across datasets

**Fig. 2:**
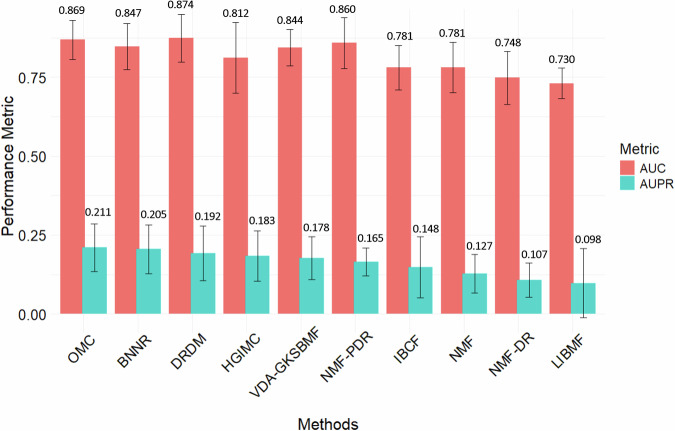
Summary of overall performance for each method across all datasets. Bars and values represent the average of median AUC and AUPR for each DR method across all datasets, with error bars indicating the standard deviation (SD).

Figure 2 presents the average performance (AUC and AUPR) of each DR method across all datasets, including the corresponding SD, ordered by AUPR from left to right. Six first methods, dominated by the MC-based approaches and DRDM have significantly higher average AUPR than MF-based methods. Particularly, OMC (0.211) achieves the highest average AUPR, slightly greater than BNNR (0.205), followed by DRDM (0.192), HGIMC (0.183), VDA-GKSBMF (0.178) and NMF-PDR (0.165). Among these top six methods, DRDM obtains the highest AUC value (0.874), followed by OMC (0.869), NMF-PDR (0.860), BNNR (0.847), VDA-GKSBMF (0.844) and HGIMC (0.812). DRDM gains the highest AUC (0.874), but its AUPR (0.192) is slightly lower than that of OMC (0.211) and BNNR (0.205). The SD values (see detail in Supplementary Table S3) for AUPR are relatively high (>0.07) for OMC, BNNR, DRDM, HGIMC, IBCF, and LIBMF, indicating greater variability across datasets, while MF-based methods, VDA-GKSBMF, NMF-PDR, NMF, NMF-DR, show more stable performance. Additionally, DRDM, HGIMC, NMF-PDR, NMF-DR and BNNR have high SD values for AUC (>0.07), suggesting lower stability in their predictions.

NMF-PDR significantly better than almost other MF-based methods in both AUC (0.860) and AUPR (0.165), except that its AUPR is slightly lower than that of VDA-GKSBMF (0.178). IBCF (0.148) is better than standard NMF (0.127) in AUPR while their AUCs are similar. In our evaluation, NMF-DR, the MF-based method designed for DR, does not show better results than standard NMF. LIBMF, which performs MFon *M*_*MC*_ rather than *M*_*MF*_ as used in other MF-based methods exhibits the worst performance (AUC = 0.730, AUPR = 0.098). Additionally, its SD is relatively large compared to its mean values, especially for AUPR (>0.1), suggesting high variability across datasets.

### Dataset-specific performance across methods

**Fig. 3:**
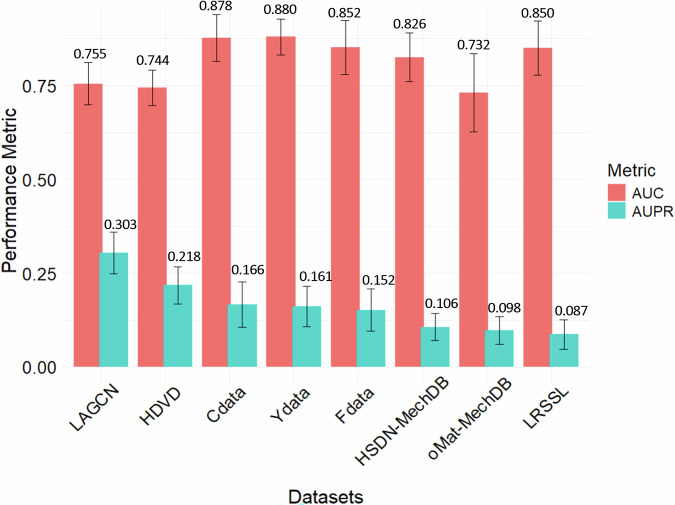
Summary of overall performance of all methods for each dataset. Bars and values represent the average median AUC and AUPR over *N* = 25 iterations for each dataset across all DR methods, with error bars indicating the standard deviation (SD).

Figure 3 shows the average performance (AUC and AUPR) along with the corresponding SD values of each dataset across all DR methods, ordered by AUPR from highest to lowest. Ydata (0.880) and Cdata (0.878) have the highest AUC values, followed by Fdata (0.852), LRSSL (0.850), and HSDN-MechDB (0.826). LAGCN (0.755), HDVD (0.744), and oMat-MechDB (0.732) have the lowest AUC scores. However, LAGCN and HDVD, despite their low AUCs, have the highest AUPR scores (0.303 and 0.218, respectively). While Ydata (0.161), Cdata (0.166), and Fdata (0.152) perform well in AUC, their AUPR values are lower. Symptom-based datasets and LRSSL show the lowest performance across all methods. The SD values are small, indicating that the results across the DR methods are consistent for each dataset, except for oMat-MechDB, which shows a higher SD for AUC as 0.1043, see detail in Supplementary Table S4.

### Impact of data sparsity on predictive performance

**Fig. 4:**
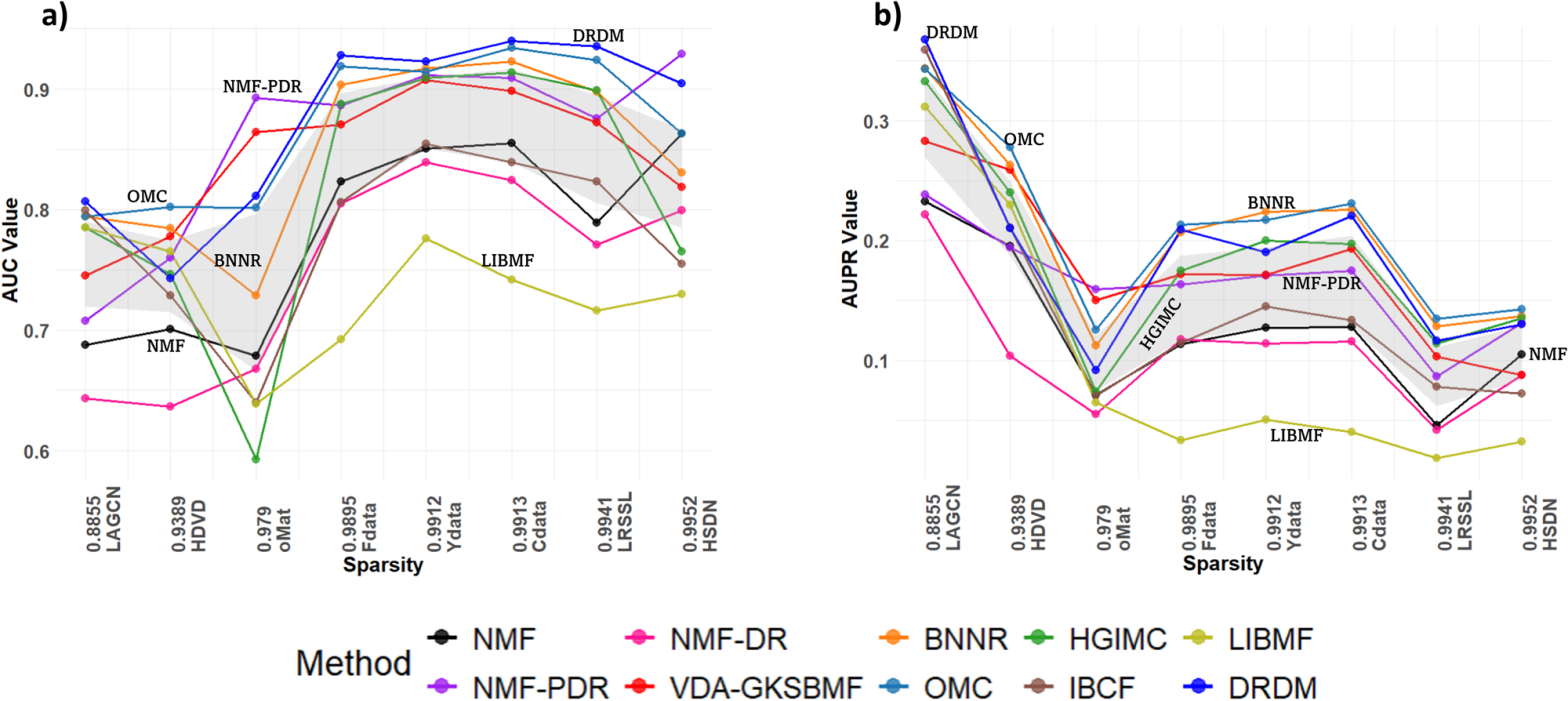
Impact of data sparsity on method performance. **a** AUC and (**b**) AUPR values for each method and each dataset, arranged in order of increasing sparsity of the dataset.

To examine the impact of sparsity on evaluation performance, Fig. 4 presents the AUC and AUPR values for each method and each dataset, ordered by increasing the sparsity of the dataset (Table 1) from left to right. The shaded regions represent the confidence intervals (CIs), indicating the variability of performance across methods.

**Table 1:** Datasets for benchmarking the drug repurposing methods

	Dataset	Drugs	Diseases	Associations	Size	Sparsity	Ref
Public datasets	HDVD	219	34	455	7446	0.9389	^59^
	LAGCN	269	598	18416	160862	0.8855	^41^
	Fdataset	593	313	1933	185609	0.9895	^60^
	Cdataset	663	409	2352	271167	0.9913	^26^
	LRSSL	763	681	3051	519603	0.9941	^61^
	Ydataset	1478	655	8448	968090	0.9912	^5^
New datasets	OMat-MechDB	89	150	271	13350	0.9797	This study
	HSDN-MechDB	1279	616	3710	787864	0.9952	This study

All drugs and diseases involve in at least one known drug-disease association. Column Size presents the number of data points in the association matrix, calculated by the multiplication of the number of drugs (column Drugs) and the number of diseases (column Diseases). Columns “Associations” presents the number of true associations. Sparsity is defined as the proportion of unknown associations, calculated by 1 - Associations/Size.

As shown in Fig. 4a, AUC values tend to be higher in datasets with greater sparsity. This is because higher sparsity (i.e., a higher number of negative instances) generally corresponds to larger sample sizes, meaning a greater total number of associations. As a result, the accuracy for true negatives improves, which can lead to a higher AUC. However, this does not necessarily imply an improvement in the prediction of true positives.

In Fig. 4b, predictive performance, as measured by AUPR, improves for datasets with lower sparsity. LAGCN, with the lowest sparsity (0.8855), achieves the highest AUPR across all methods, followed by HDVD (0.9389). In contrast, datasets with higher sparsity (>0.97) exhibit lower AUPR performance. Fdata (0.9895), Ydata (0.9912), and Cdata (0.9913) have similar sparsity levels and yield relatively similar results. LRSSL (0.9941) and HSDN-MechDB (0.9952), with the highest sparsity, show the lower AUPR values. The oMat-MechDB dataset (0.9790), despite having slightly lower sparsity, is a small dataset, which results in poor performance.

Moreover, OMC and BNNR demonstrate the highest robustness across datasets with varying sparsity, consistently maintaining high AUC and AUPR values. In contrast, DRDM shows a stable AUC trend but only moderate resilience in AUPR, while NMF-PDR maintains stable AUC but achieves lower AUPR, indicating reduced precision for positive associations. These results suggest that MC-based methods, particularly OMC and BNNR, are most suitable for real-world sparse drug-disease networks, followed by the deep learning-based DRDM.

### Impact of evaluation strategies on performance of DR models

The AUPR results of the DR methods reported in this study are significantly lower than the report from a recent benchmarking study by Li et al. 2024^8^. We realize that the discrepancy is due to differences in the CV procedures used in the two studies. Specifically, Li et al. do not perform CV on full data points, thereby avoiding the large number of negative instances during evaluation. First, they create 10 folds of the data points belonging to group “1” for cross-validation. In each CV iteration, one fold from group “1” was set aside as the test set. Then, they randomly selected a subset of data points from group “0” equal in size to the fold from group “1”. Finally, two subsets of group “0” and group “1” are combined to generate the test set for that CV iteration. Thus, the majority of data points from group “0” are ignored in this evaluation because they are never included in the test sets. This also explains for the high AUPR (>0.90) of the DR methods reported in their study. We believe our approach is more realistic and does not suffer the overestimation for DR methods.

To illustrate, Table 2 reports the comparison of different evaluation approaches on three gold standard datasets including Fdata, Cdata, and Ydata using the top three methods: OMC, BNNR, and HGIMC. The AUC and AUPR values reported for other studies are extracted from their original articles in^8^ and are labeled as ’Li et al. 2024’. Results labeled as ’This study’ correspond to outputs from our study. Overall, both AUC and AUPR values from Li et al. 2024 are consistently higher than those obtained in our study across all datasets. Notably, the AUPR values in our study (all <0.24) are significantly lower than those in Li et al. 2014 (all >0.93), highlighting the substantial differences between the two evaluation approaches.

**Table 2:** Comparison of evaluation performance results using two different evaluation methods

Methods		BNNR			OMC			HGIMC		
Datasets		Fdata	Cdata	Ydata	Fdata	Cdata	Ydata	Fdata	Cdata	Ydata
AUC	Li et al. 2024	0.9362	0.9501	0.9511	0.9465	0.9593	0.9503	0.9182	0.9415	0.9499
	This study	0.9031	0.9235	0.9168	0.9196	0.9345	0.9143	0.8880	0.9130	0.9096
AUPR	Li et al. 2024	0.9515	0.9629	0.9649	0.9567	0.9680	0.9649	0.9365	0.9541	0.9607
	This study	0.2051	0.2254	0.2237	0.2149	0.2321	0.2128	0.1750	0.1966	0.2001

Results of row “Li et al. 2024" are obtained from the evaluation method in^8^, where the test set consists of a small, balanced subset of positive associations (1s) and an equal number of negative associations (0s). AUC and AUPR of row “This study" are derived from our evaluation approach (detailed in Section 4.8), which considers the entire dataset for the evaluation. The comparison highlights significant differences in performance metrics (AUC and AUPR) for Fdata, Cdata, and Ydata using the top three methods, OMC, BNNR, and HGIMC, under both evaluation strategies.

Another key difference is in the approach to calculating AUC and AUPR. Many studies, including the research of Li et al. pool the predicted results of all diseases together for the calculation. In contrast, we focus on identifying the top-ranked predictions for each disease, prioritizing drugs with the highest association scores in the prediction score matrix. We argue that our approach is more practical for identifying and prioritizing repurposed drugs for individual diseases. In some studies^39^, the reported AUPR values are also significantly higher than ours. However, the details of their evaluation approaches are not always clearly described, making result reproducibility challenging.

### Impact of using multiple similarity measurements on performance of DR models

Some DR methods, such as HGIMC, enable the integration of multiple drug-drug or disease-disease similarity matrices, which are derived from various drug- or disease-related information, before being used in their prediction models. Similarly, DRDM provides flexibility to operate with either single or multiple similarity matrices, as reported in its original study. For example, different drug-related features, such as chemical structures (ChemS), anatomical therapeutic chemical (ATC) codes (AtcS), side effects (SideS), drug-drug interactions (DDIS), and target profiles (TargetS), can be integrated to construct the final drug-drug similarity matrix. Likewise, disease-disease similarity can be derived from a combination of features, such as disease phenotypes (PhS) and disease ontology (DoS). In these two methods, DRDM and HGIMC, the final drug-drug and disease-disease similarity matrices are typically obtained by averaging the individual similarity measures for each, respectively.

**Fig. 5:**
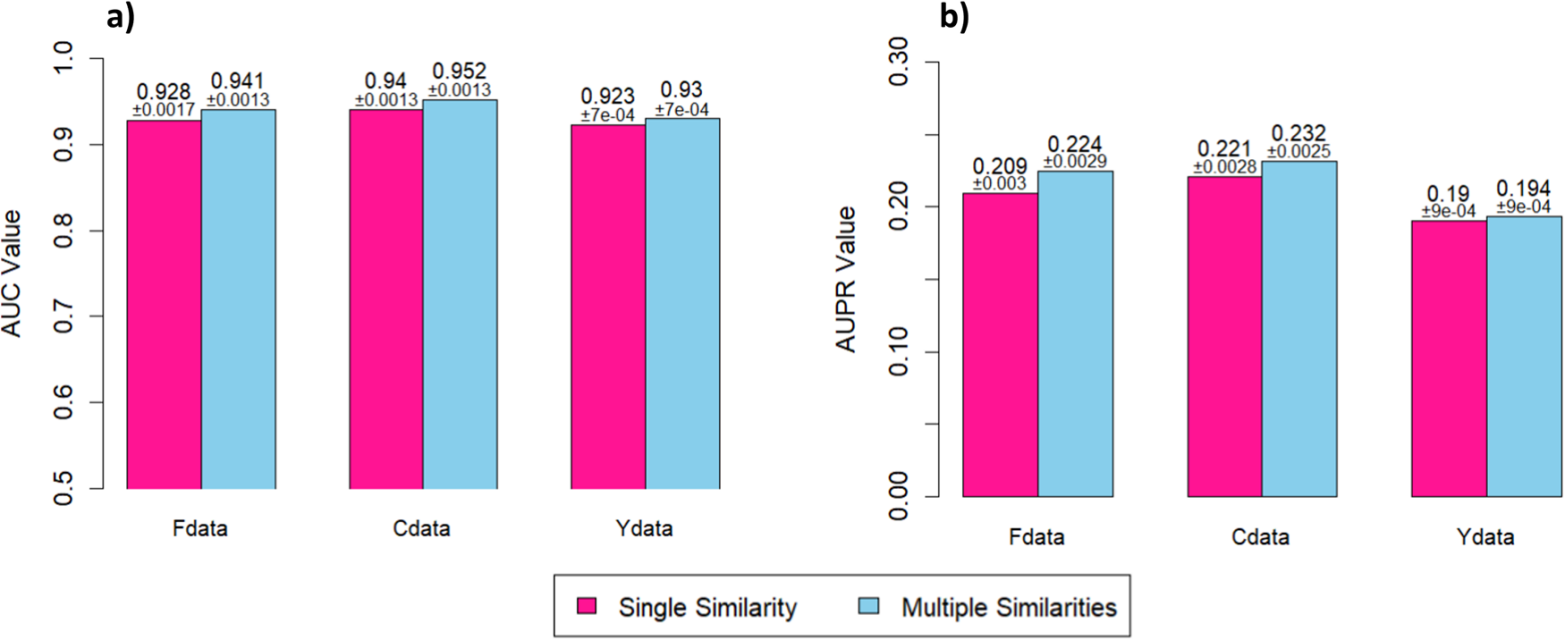
DRDM model performance using individual similarities and multi-similarities in 10-fold cross-validation. Median AUC and AUPR over 25 runs for each model, with corresponding standard deviations, are shown: **a** AUC comparison. **b** AUPR comparison.

**Fig. 6:**
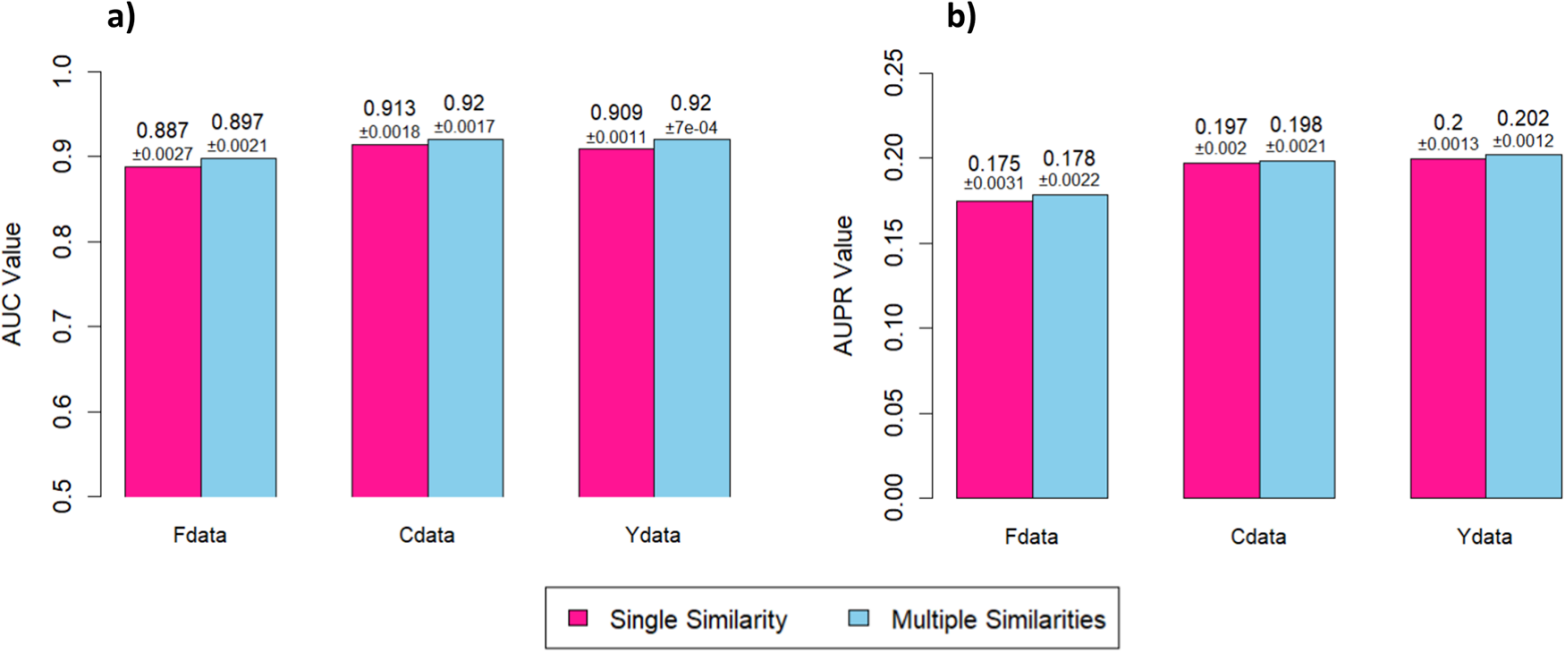
HGIMC model performance using individual similarities and multi-similarities in 10-fold cross-validation. Median AUC and AUPR over 25 runs for each model, with corresponding standard deviations, are shown: **a** AUC comparison. **b** AUPR comparison.

This section evaluates the performance of DRDM and HGIMC using the multiple similarity mode (default setting for HGIMC and also applicable to DRDM) compared to the single similarity mode (used in this study). In the single similarity mode, only ChemS for drugs and PhS for diseases are used. We apply DRDM and HGIMC in both modes to three commonly used DR datasets: Fdata, Cdata, and Ydata. As shown in Fig. 5 for DRDM, and Fig. 6 for HGIMC, the two modes exhibit only slight differences in performance, with the multiple similarity mode yielding a marginally better outcome. Both HGIMC and DRDM models demonstrate high stability, with SD ≤0.0031 for both AUC and AUPR across single and multiple similarity settings.

The slight performance difference between the single similarity mode and the multiple similarity mode can be mainly attributed to the high degree of redundancy among the different similarity measures. To examine this, we analyze the correlations between the single and integrated similarity modes for both diseases and drugs (see Supplementary Figs. S3–5). The disease similarity matrices of the two modes show a very high correlation (0.93 in all three datasets), indicating that integrating multiple similarity measures do not introduce substantial new information. In contrast, the drug similarity matrices exhibit a lower correlation (0.38–0.57), suggesting that drug similarities vary more across different measures, which may account for the observed performance gap. In addition, the overall effect of similarity integration depends on the chosen integration strategy. The current averaging approach assigns equal weights to all similarity types, which may not effectively handle redundancy among drug similarity measures. A more sophisticated integration strategy (e.g., weighting for similarity types) could potentially yield further improvements and require further investigation.

### Comparison in computational time

The computation time for each method on a dataset depends on both method’s algorithm and the dataset size. For comparison, we report the computational time for 10-fold cross-validation of each method across the datasets. All methods are executed on a personal computer running Microsoft Windows 10 Enterprise, with an Intel® Core™ i7 2.80 GHz processor and 32 GB RAM, using a single CPU (no parallel processing). The computation time is measured from the start of execution until completion.

**Fig. 7:**
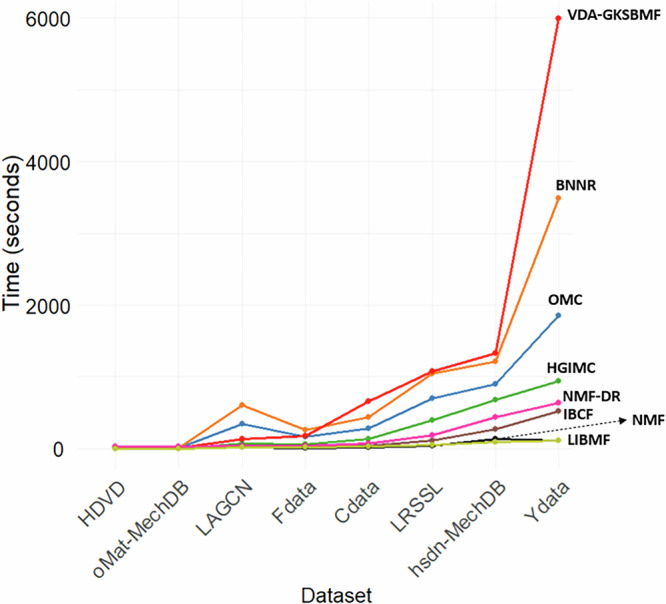
Computational time running for each method on each dataset. The values represent the runtime (in seconds) for each method applied to a specific dataset.

Figure 7 reports the computational performance of the DR methods across datasets. In this plot, the y-axis is the computational time measured by seconds. The x-axis represents the datasets, ordered from left to right by increasing size of their association matrix (*n* × *m*). In general, the computational time of the methods is proportional to the size of the dataset. Among them, NMF-PDR, VDA-GKSBMF, BNNR and OMC demand most computational time.

For the Ydata dataset, the largest in this study, NMF-PDR requires up to 5.5 h to complete. This is due to its permutation procedure, where NMF is executed multiple times. Additionally, DRDM requires more than 10 h to finish. This extended runtime is primarily due to the computational complexity of the dual-view contrastive learning and adaptive debiasing modules. To prevent it from dominating and distorting the plot, we have excluded them from the line plot in Fig. 7. Although these methods achieve strong predictive performance, their longer runtime may limit scalability, especially in large-scale DR tasks. This reflects the inherent trade-off between model performance and computational efficiency. To mitigate this issue, optimization strategies, such as GPU acceleration, parallel or distributed computing, and algorithmic simplification can be employed to improve efficiency without substantially compromising predictive accuracy.

IBCF and LIBMF from the Recommenderlab package demonstrate the fastest performance. Detailed computational times for each method and dataset are reported in Supplementary Table S5.

## Discussion

This study performs a rigorous evaluation of ten advanced computational DR methods across eight datasets. We also introduce two new symptom-based drug-disease association datasets, and propose a new NMF-PDR algorithm that significantly enhances the performance of the standard NMF performance across all DR datasets.

There are no method dominant in all datasets, but top-ranked methods are OMC, BNNR, DRDM, HGIMC, VDA-GKSBMF, and NMF-PDR, followed by IBCF, NMF, NMF-DR, and LIBMF. Overall, OMC, an improved version of BNNR^33^, shows superior performance in most performance metrics. Moreover, DRDM ranks among the top methods in both AUC and AUPR demonstrating that deep learning can perform effectively and that DRDM is a good candidate for DR studies.

However, our method ranking results differ from the recent benchmark study^8^, where they report top performing methods in decreasing order as HGIMC, BNNR, VDA-GKSBMF, OMC, and NMF-DR. The discrepancy likely results from variations in both cross-validation approach and evaluating dataset. Li et al.^8^ evaluate DR methods using datasets with varying structures. For example, HGIMC is applied using multi-similarity measures for drugs and diseases, whereas other methods are evaluated using single-similarity data. Additionally, HGIMC and NMF-DR are tested on only four datasets, OMC on eight, while BNNR and VDA-GKSBMF are tested on 11 datasets. In contrast, our study focuses on evaluation of DR methods on the same datasets with the same condition of using single similarity measure for both drugs and diseases. Using this approach, we can highlight the strength of algorithm in the DR methods. However, it is noted that this approach ignores the strength of the methods in integrating multiple similarity measurements.

Although the proposed NMF-PDR method does not outperform the top-performing method, OMC, it surpasses other NMF-based approaches, highlighting the benefits of incorporating permutation testing to enhance the NMF algorithm. This suggests that NMF-PDR could be valuable in other applications where NMF has been widely applied. Exploring its performance in such contexts will be our future work.

Besides the heterogeneous network-based approaches in this study, other recent computational approaches, for example, modern artificial intelligence^40^ also demonstrate promising results. However, these DR methods require different data input formats, therefore we exclude them from our evaluation.

In summary, this study provides a benchmarking analysis of ten advanced heterogeneous network-based DR methods across eight diverse datasets. We demonstrate that methods, such as OMC, BNNR, DRDM, HGIMC, VDA-GKSBMF, and NMF-PDR achieve high performance across various evaluation metrics. Our findings enhance the understanding of DR methods, introduce additional datasets for evaluation, and facilitate the DR research.

## Methods

This section will introduce 1) the heterogeneous drug-disease network for DR, 2) the competing DR methods (Table 3) used in this study, 3) the collection of DR datasets, and 4) the evaluation approach to benchmark the DR methods on the datasets. The details are described in the following sections.

**Table 3:** Drug repurposing methods based on heterogeneous networks in this study

Methods		Algorithms	Adjacency Matrix	Language	Ref
MC-approaches	BNNR	Nuclear norm regularization, ADMM	*M*_*MC*_ (Eq.(2))	Matlab	^31^
	OMC	KNN, Nuclear norm minimization, ADMM	${M}_{OMC}^{r}$, ${M}_{OMC}^{d}$ (Eq.(3))	Matlab	^33^
	HGIMC	HGBI, bounded matrix completion, Gaussian radial basis, ADMM	Association Matrix *M*_*rd*_	Matlab	^4^
MF-approaches	NMF	Non-negative matrix factorization, Gradient Descent, Multiplicative update rules	*M*_*MF*_ (Eq.(1))	R	^34^
	NMF-PDR	Non-negative matrix factorization, Gradient Descent, Multiplicative update rules, Permutation	*M*_*MF*_ (Eq.(1))	R	^34^
	NMF-DR	Non-negative matrix factorization, Similarity Network Fusion	*M*_*MF*_ (Eq.(1))	Matlab	^27^
	VDA-GKSBMF	Gaussian kernel similarity, bilinear matrix factorization, ADMM	Association Matrix *M*_*rd*_	Matlab	^32^
RS approaches	IBCF	collaborative filtering, recommenderlab package	*M*_*MC*_ (Eq.(2))	R	^53^
	LIBMF	collaborative filtering, negative matrix factorization, recommenderlab package	*M*_*MC*_ (Eq.(2))	R	^36,37,53^
DL approach	DRDM	Graph Neural Network, LightGCN, Adaptive debiasing mechanism, Dual-view contrastive learning	*M*_*DRDM*_ (Eq. (11)), ${\widehat{M}}_{DRDM}$ (Eq. (12))	Python	^22^

### Heterogeneous drug-disease network for DR

**Fig. 8:**
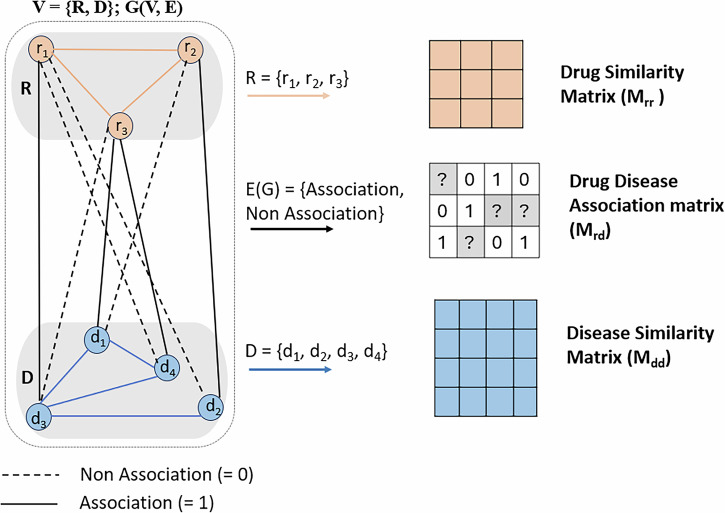
Heterogeneous Drug-Disease Network.

The heterogeneous drug-disease network^27,28^ presents the drug-drug, drug-disease and disease-disease relations in a given set of drugs and diseases, Fig. 8. We define a set of drugs $R=\left({r}_{1},{r}_{2},...,{r}_{n}\right)$ with *n* as the number of drugs and a set of diseases $D=\left({d}_{1},{d}_{2},...,{d}_{m}\right)$, with *m* as the number of diseases.

In graph presentation, the edge between drugs *r*_*i*_ and *r*_*j*_ is weighted by similarity between two drugs. Similarly, the edge between diseases *d*_*i*_ and *d*_*j*_ is weighted by similarity between two diseases. Besides, the drug-disease associations can be modeled as a bipartite graph $G\left(V,E\right)$, where $V\left(G\right)=\{R,D\}$, $E\left(G\right)\subseteq R\times D,E\left(G\right)=\{{e}_{ij},\,\mathrm{edg}e\,\mathrm{between}\,\mathrm{drug}\,{r}_{i}\,\mathrm{and}\,\mathrm{disease}\,{d}_{j}\}$. If drug *r*_*i*_ and disease *d*_*j*_ are associated, the weight of the edge between *r*_*i*_ and *d*_*j*_ is initially set to 1, otherwise, it is initially set to 0^27,31^.

In matrix presentation, the heterogeneous drug-disease network for DR is supported by three key types of input matrices. First, the drug-drug similarity matrix *M*_*rr*_ captures similarity between drugs, which is calculated using a single or multiple characteristics of drugs, such as chemical structures, associated genes, etc. Second, the disease-disease similarity matrix *M*_*dd*_ represents the relationships between diseases. It can be calculated using various approaches, such as semantic similarity, clinical features, molecular signatures like omics data. Finally, the drug-disease association matrix *M*_*rd*_ is often a binary representation of drug-disease relationship, where 0 indicates the absence or no association, and 1 denotes a true association. The information of the association is usually gathered from drug and disease databases.

For convenience, in this study, we classify each drug-disease pair into either the “1” group (indicating a true association) or the “0” group (indicating no association).

### Drug-disease adjacency matrix

Several methods including HGIMC and VDA-GKSBMF directly use the drug-disease association matrix, *M*_*rd*_, however, many methodologies construct a drug-disease adjacency matrix to integrate information from *M*_*rr*_, *M*_*dd*_, and *M*_*rd*_ for DR. There are multiple ways to construct the adjacency matrix from these similarity matrices. In this study, we describe three adjacency matrices that are commonly used in MC and MF methods for DR.

**Fig. 9:**
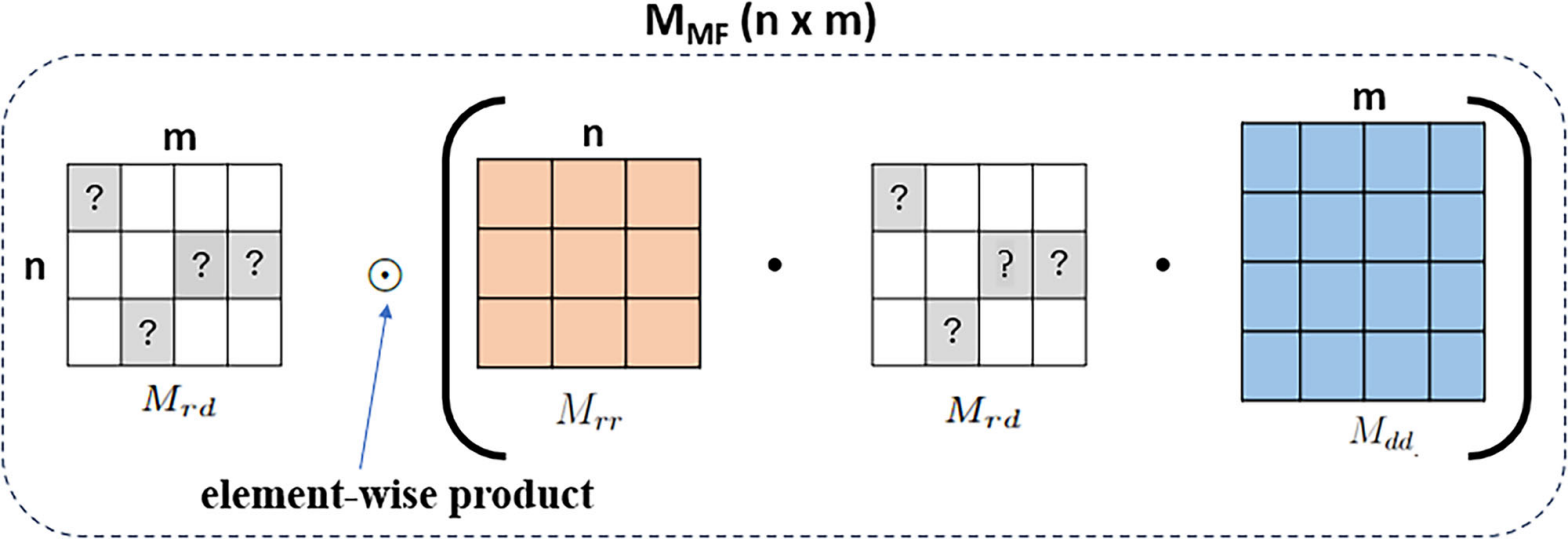
Drug-disease adjacency matrix *M*_*MF*_ constructed by the Hadamard product of *M*_*rr*_, *M*_*rd*_, and *M*_*dd*_.

The first approach, used in MF methods, is illustrated in Fig. 9, where the adjacency matrix *M*_*MF*_ is defined in Equation (1):

1 $${M}_{MF}={M}_{rd}\odot \left({M}_{rr}\cdot {M}_{rd}\cdot {M}_{dd}\right),$$

where ⊙ denotes the Hadamard product (element-wise multiplication). Thus, the adjacency matrix *M*_*MF*_ and the drug-disease association matrix *M*_*rd*_ have the same dimensions. However, in *M*_*MF*_, drug-disease pairs of the “1” group (*M*_*rd*_ = 1) are weighted based on drug-drug and disease-disease similarities in *M*_*MF*_. For drug-disease pairs of the “0” group (*M*_*rd*_ = 0), their values in the adjacency matrix remain unchanged (i.e., 0).

The adjacency matrix is then normalized to the range [0,1] by applying ${M}_{MF}=\frac{{M}_{MF}}{\max \left({M}_{MF}\right)}$, following the fundamental principles of weighted MF^27^. In this study, this adjacency matrix will be used for the NMF, NMF-PDR, NMF-DR models.

**Fig. 10:**
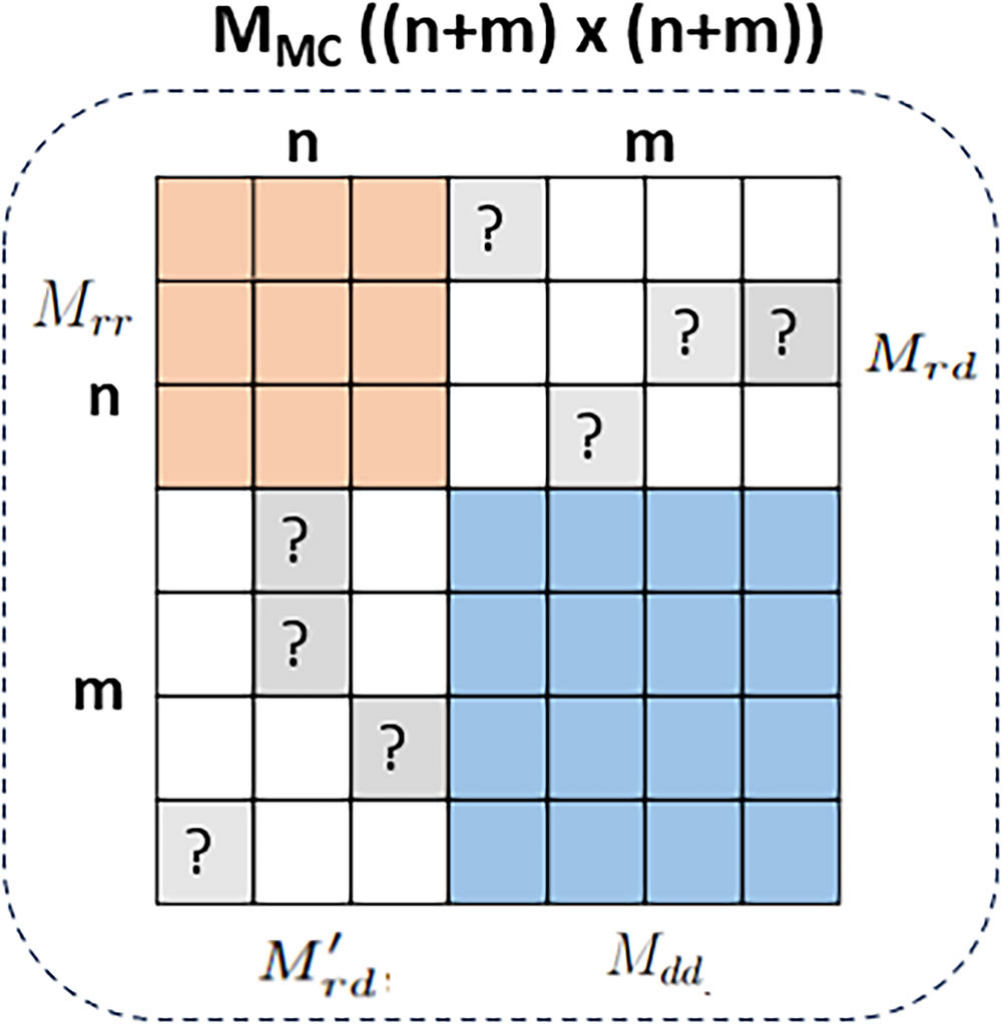
Drug-disease adjacency matrix *M*_*MC*_ as a block matrix constructed from *M*_*rr*_, *M*_*dd*_, ${M}_{rd}^{{\prime} }$ and *M*_*rd*_.

Figure 10 presents the second approach for constructing the adjacency matrix, used in MC methods, where *M*_*MC*_ is a block matrix of four submatrices including *M*_*rr*_, *M*_*rd*_, ${M}_{rd}^{{\prime} }$ and *M*_*dd*_, as formalized in Equation (2):

2 $${M}_{MC}=\left[\begin{array}{cc}{M}_{rr} & {M}_{rd}\\ {M}_{rd}^{{\prime} } & {M}_{dd}\end{array}\right]$$

where ${M}_{rd}^{{\prime} }$ is the transposed of *M*_*rd*_. The diagonal submatrices *M*_*rr*_ and *M*_*dd*_ are dense, representing the similarity between drugs and diseases, respectively. The unknown entries are only presented in the off-diagonal submatrices *M*_*rd*_ and ${M}_{rd}^{{\prime} }$, representing the unknown associations to be predicted. By incorporating both direct and indirect connections between drugs and diseases, this extended matrix structure improves the ability to predict novel associations. In this study, this adjacency matrix is used as the input for the BNNR model and two RS tools including IBCF and LIBMF.

The final construction approach for the adjacency matrix is presented in Equation (3). In this approach, separate adjacency matrices are used: one for drug-drug similarity (*M*_*rr*_), referred to as the drug-side network (${M}_{OMC}^{r}$), and one for disease-disease similarity (*M*_*dd*_), referred to as the disease-side network (${M}_{OMC}^{d}$). This approach is specifically utilized in the OMC algorithm.

3 $${M}_{OMC}^{r}=\left[\begin{array}{c}{M}_{rr}\\ {M}_{rd1}\end{array}\right]\,\,\,{\rm{and}}\,\,\,{M}_{OMC}^{d}=\left[\begin{array}{c}{M}_{dd},{M}_{rd2}\end{array}\right],$$

where *M*_*rd*1_ and *M*_*rd*2_ are updated versions of the association matrix *M*_*rd*_ by a k-nearest neighbors (KNN) algorithm in the preprocessing step, ensuring that each disease and each drug has at least one association with drugs and diseases, respectively. The details are referred to the original study^33^.

### MF based methods

This section introduces four MF-based DR methods used in the evaluation. We begin with a description of the NMF algorithm, followed by a brief summary of two publicly available MF-based DR methods, NMF-DR and VDA-GKSBMF. Finally, we propose NMF-PDR, a novel permutation approach for the NMF method.

#### Non-negative MF (NMF)

NMF is an ideal choice for DR models due to its ability to reveal hidden patterns and relationships within datasets, which is particularly important for uncovering unknown associations in the drug-disease association matrix^27,41^. NMF aims to decompose a given non-negative matrix *V* ∈ *R*^*n*×*m*^ into the product of two non-negative matrices *W* ∈ *R*^*n*×*r*^ and *H* ∈ *R*^*r*×*m*^ such that

4 $$V\approx WH,$$

where rank *r* is the dimension of drug feature and disease feature in the lower-rank spaces. This rank parameter determines the size of the new sub-matrices and is the primary parameter of NMF.

The objective of the NMF algorithm is to minimize the dissimilarity $J\left(V| | WH\right)$ between the original matrix *V* and its approximation *WH* using a cost function,

5 $$\mathop{\min }\limits_{W,H\ge 0}J\left(V| | WH\right).$$

Traditionally, $J\left(V| | WH\right)$ is based on the least squares formula as,

6 $$\mathop{\min }\limits_{W,H\ge 0}| | V-WH| {| }^{2}.$$

NMF was popularized by Lee and Seung due to their significant contribution of a simple yet effective algorithmic procedure as introduced in^34^, which identified the multiplicative update rules for decomposed matrices,

7 $${H}_{ij}={H}_{ij}\frac{{\left({W}^{T}V\right)}_{ij}}{{\left({W}^{T}WH\right)}_{ij}}\,{W}_{ki}={W}_{ki}\frac{{\left(V{H}^{T}\right)}_{ki}}{{\left(WH{H}^{T}\right)}_{ki}}.$$

In the context of DR, the adjacency matrix *M*_*MF*_ from Equation (1) acts as the input matrix *V* of the NMF model to predict new drug-disease associations.

Selecting the value of rank, *r* is a critical step that significantly affects the results of the NMF model. An excessively small rank can lead to the loss of valuable features, while an overly large rank could model noise. Several methods for rank selection have been proposed in the literature^27,42–44^. In this study, we used an SVD-based approach to determine the rank of the NMF and NMF-PDR models by assessing the explained cumulative variance^44^. Particularly, SVD decomposes the matrix into three matrices: *V* = *A*Σ*B*^*T*^, where *A* contains the left singular vectors, Σ is a diagonal matrix containing the singular values and *B* contains the right singular vectors. The cumulative explained variance (CEV) is calculated as

8 $${{\rm{CEV}}}_{k}=\frac{{\sum }_{i=1}^{k}{\sigma }_{i}^{2}}{{\sum }_{i=1}^{n}{\sigma }_{i}^{2}}$$

where *σ*_*i*_ represents the singular value *i*, *k* denotes the current component and *n* is the total number of singular values. The rank *r* is selected where CEV_*r*_ reaches or exceeds a specified threshold. We set a threshold of 0.90 to capture 90% of the total variance in the original data.

In the implementation, the NMF method is developed in R using the Lee-Seung update rules, as shown in Equation (7). The rank *r* is determined by an SVD-based approach. It iteratively refines the factor matrices *W* and *H* to minimize reconstruction error ∣∣*V* − *WH*∣∣^2^ until the convergence criteria are met.

#### Non-Negative MF-based DR (NMF-DR)

NMF-DR^45^ improve the DR performance through optimizing the rank selection, initial values, prediction step of NMF. The method begins by building a heterogeneous drug-disease association network, as illustrated in Fig. 9 and defined in Equation (1), through the integration of drug and disease similarity networks. Different from the standard NMF, the NMF-DR methodology involves three key steps: (1) selecting a suitable factorization rank *r* using the minimum description length (MDL) criterion; (2) initializing the factor matrices based on a SVD-based method. (3) predicting drug-disease association by combination of the NMF method with an accelerated hierarchical alternating least squares (A-HALS) algorithm.

#### Gaussian kernel similarity bilinear MF (VDA-GKSBMF)

VDA-GKSBMF^32^ is a MF-based method which employs Gaussian kernel similarity and bilinear MFn to explore potential virus-drug associations for SARS-CoV-2. Particularly, VDA-GKSBMF applies Gaussian kernel similarity to the association matrix to enhance both virus and drug similarity, which improves the predictive capacity of bilinear MF^32^. This approach identifies new antiviral drugs by predicting unknown virus-drug associations and optimizing the model with the alternating-direction multiplier method (ADMM). Although originally designed for drug-virus associations, VDA-GKSBMF can also be applied to other drug-disease association datasets, as used in this study.

#### Permutation-based NMF approach for DR (NMF-PDR)

In this section, we introduce NMF-PDR, a proposed permutation approach for NMF in DR. NMF-PDR assumes that the drug-disease associations are not random, but are instead driven by biological and/or chemical relationships. Therefore, the prediction from NMF on the true drug-disease association matrix should be significantly higher than those from a random association matrix. To consider this, NMF-PDR employs a permutation-based approach to generate random association matrices, compares the NMF-predicted values from the adjacency matrix *M*_*MF*_ (Equation 1) of the true associations against these of random matrices (null distribution), and utilizes this information for DR.

First, we collect the group labels of drug-disease pairs based on the true association matrix *M*_*rd*_: the “1” group (indicating a true association) and the “0” group (indicating no association). Then, for drug-disease pairs of group “1”, we permute their values in the adjacency matrix (*M*_*MF*_) *K* times (e.g, *K* = 100) to create *K* observed adjacency matrices. Thus, the set of true associations in the observed adjacency matrices are the same, but their values are swapped, reflecting the variations in drug-drug and disease-disease similarity. The standard NMF method is then applied to the observed adjacency matrices to obtain *K* predicted matrices of observed values (*O*_1_).

Next, NMF-PDR permutes the adjacency matrix *K* times by rows, columns, and both to create random associations between drugs and diseases, then applies NMF to these permuted matrices. This process creates three types of null distributions: *R*_0_ (row permutations), *C*_0_ (column permutations), and *B*_0_ (both row and column permutations), respectively.

Finally, we apply Wilcoxon tests to compare *O*_1_ against *R*_0_, *C*_0_, and *B*_0_, then combine three test statistics by using Stouffer’s Z-score method, as the final predicted values of NMF-PDR. The Wilcoxon non-parametric rank test is selected as it yields better results for most datasets compared to the other tests, such as t-test, KS-test in this study (Supplementary Table S6).

Details of the algorithm of NMF-PDR are provided in the Supplementary document.

### MC methods

We summarize three MC-based methods used in this study including BNNR^31^, OMC^33^ and HGIMC^4^.

#### Bound nuclear norm regulalization (BNNR)

BNNR ultilizes nuclear norm minimization with a bounded constraint to predict drug-disease associations in the range of $\left[0,1\right]$. It integrates drug-drug, drug-disease, and disease-disease networks into a heterogeneous network, presented as the adjacency matrix *M*_*MC*_ (Equation (2)) which serves as the input for the MC.

Since direct rank minimization is NP-hard^31,46,47^, the rank optimization problem can be relaxed to a nuclear norm minimization^48,49^:

9 $$\mathop{\min }\limits_{X}| | X| {| }_{* }\,\,\,{\rm{s.t}}\,\,{P}_{\Omega }\left(X\right)={P}_{\Omega }\left(M\right),$$

where ∣∣*X*∣∣_*_ denotes the nuclear norm of *X*, defined as the sum of its singular values. To ensure predictions remain within $\left[0,1\right]$, BNNR imposes additional bounds:

10 $$\mathop{\min }\limits_{X}| | X| {| }_{* }+\frac{\alpha }{2}| | {P}_{\Omega }\left(X\right)-{P}_{\Omega }\left(M\right)| {| }^{2}\,\,{\rm{s.t}}\,\,0\le X\le 1,$$

where *α* is parameter balancing the nuclear norm and the error term. The regularization term $| | {P}_{\Omega }\left(X\right)-{P}_{\Omega }\left(M\right)| |$ controls how much noise the model tolerates by bounding the error between the observed and predicted values, ensuring robustness to noise in the input data.

#### Overlap MC (OMC)

OMC extends BNNR by incorporating multilayered network data, improving prediction accuracy and robustness. In our evaluations, we focus on a bi-layer network of drugs and diseases. It includes a preprocessing step that uses K-Nearest Neighbors (KNN) to impute a few novel associations for de novo drugs or diseases (i.e, no associations exist or the drugs or diseases). It then constructs separate updated drug-disease association matrices for drugs *M*_*rd*1_ and diseases *M*_*rd*2_. Instead of a large heterogeneous matrix, OMC integrates integrates two separate networks as in (Equation (3)), then applies BNNR separately to each. The final predictions are averaged to improve reliability.

#### Heterogeneous graph inference with MC (HGIMC)

Similar to OMC, HGIMC also utilizes BNNR, but combines with the guilt-by association principle of HGBI^50,51^. It refines drug and disease similarity matrices using Gaussian radial basis functions (GRB) before applying bounded MC (BMC) with the optimization equation (Equation (10)) of BNNR model to impute high confidence drug-disease associations. This step enriches the edges connecting drug and disease networks. Finally, it integrates the updated drug and disease similarity matrices with the updated drug-disease association matrix to predict the unknown associations.

Originally, HGIMC was designed to integrate multiple similarity measures for drugs and diseases. However, since this study focuses on methods using a single similarity measure, we apply HGIMC in single-similarity mode. Our analysis also shows minimal performance differences between using single and multiple similarity measures (Section 2.5).

### Other methods from recommendation system

A recommendation system (RS) aims to predict user preferences and suggest relevant items based on historical data. It is widely used in e-commerce, streaming services, and social media. DR and recommendation systems share a common mathematical foundation: predicting missing associations in a matrix.

In this study, we select two widely used recommendation system methods including item-based collaborative filtering (IBCF)^35^ and library for parallel MF in shared memory systems (LIBMF)^36,37,52^ for application in DR. Briefly, LIBMF improves speed and performance by applying a learning-rate schedule for stochastic gradient methods to MF^36,37^. IBCF predicts user preferences by analyzing item-to-item similarities rather than user similarities. It first constructs an item-item similarity matrix using metrics like cosine similarity or Pearson correlation, based on user interaction patterns. For a given item, IBCF identifies similar items and predicts a user’s preference by aggregating ratings from these related items. Both methods are implemented in the *recommenderlab* R-package^53^.

### Deep learning framework with a debiasing mechanism (DRDM)

DRDM^22^ is a graph neutral network-based framework designed to improve drug-disease association prediction by addressing popularity bias and enhancing representation robustness through dual-view contrastive learning. The model operates on two complementary graphs. The association graph *M*_*DRDM*_ is derived from the drug-disease association matrix *M*_*rd*_ as

11 $${M}_{DRDM}=\left(\begin{array}{cc}0 & {M}_{rd}\\ {M}_{rd}^{T} & 0\end{array}\right),$$

and the neighbor graph ${\widehat{M}}_{DRDM}$ captures the drug-drug and disease-disease similarities as:

12 $${\widehat{M}}_{DRDM}=\left(\begin{array}{cc}{\widehat{M}}_{d} & 0\\ 0 & {\widehat{M}}_{r}\end{array}\right).$$

Here ${\widehat{M}}_{d}$ and ${\widehat{M}}_{r}$ are obtained by retaining only the top-*K* most similar pairs for diseases and drugs, respectively, to filter out irrelevant connections,

$${\widehat{M}}_{d,(i,j)},=\left\{\begin{array}{ll}{{M}_{dd}}_{(i,j)}, & \,\mathrm{if}\,\,{{M}_{dd}}_{(i,j)}\,\mathrm{belongs\; to\; top}\,-K\,\,\mathrm{values}\\ 0, & \,\mathrm{otherwise}\end{array}\right.$$

$${\widehat{M}}_{d,(i,j)},=\left\{\begin{array}{ll}{{M}_{rr}}_{(i,j)}, & \,\mathrm{if}\,\,{{M}_{rr}}_{(i,j)}\,\mathrm{belongs\; to\; top}\,-K\,\,\mathrm{values}\\ 0, & \,\mathrm{otherwise.}\end{array}\right.$$

where filter threshold *K* is a fixed hyperparameter for each dataset.

The core strategies of DRDM combine multiple complementary approaches to improve prediction accuracy and robustness. First, multi-view feature fusion integrates various similarity measures into unified node embeddings using a learnable attention mechanism, capturing diverse relationships among drugs and diseases. Second, de-biased graph convolution uses a LightGCN^54^ as the backbone model, where neighbor messages are rescaled based on node popularity, reducing the dominance of highly connected nodes while enhancing representations for long-tail entities. Third, consistency regularization employs dual-view contrastive learning to align embeddings across the association and neighbor graphs, further improving robustness and generalization. Together, these strategies enable DRDM to generate more accurate and generalizable drug-disease predictions while mitigating the effects of dataset biases.

### Datasets

#### Public datasets

A total of six DR datasets (Table 1, Supplementary Section 1.5) from a recent study^8^, available at https://zenodo.org/records/8357512 are collected to evaluate the performance of the DR methods. Each data contains three matrices of drug-drug similarity, disease-disease similarity, and drug-disease association. Among these, Fdata, Cdata, and Ydata are widely recognized as gold standard datasets for benchmark DR methods^8^. The drugs in most of these datasets come from the DrugBank database^55^, while diseases are derived from the Online Mendelian Inheritance in Man (OMIM) database^56^, the Comparative Toxicogenomics Database (CTD)^57^ and MeSH. All these datasets exhibit high sparsity. Specifically, Fdata, Cdata, LRSSL, and Ydata have sparsity values greater than 0.98, while HDVD and LAGCN have slightly lower sparsity levels of 0.93 and 0.88, respectively.

#### Symptom-based datasets

We construct two new DR datasets including *oMat-MechDB* and *HSDN-MechDB*, summarized in Table 1. Both datasets utilize MechDB, the recent largest curated database^38^ from the Drug Mechanism Database project to collect drug-disease associations. Additionally, they both construct drug-drug similarity matrix by the Tanimoto coefficient computed based on the chemical structures of drug pairs. The chemical structures of drugs are collected from DrugBank database. A key difference between these and the public datasets is that disease-disease similarity is calculated using clinical symptoms. The rationale is that diseases with overlapping symptoms are likely to share common biological pathways. This approach is typically useful for rare diseases, as many are primarily identified by their symptoms, while research on the diseases and their molecular mechanisms remains limited.

For the two symptom-based datasets, oMat-MechDB focuses on rare diseases with the disease symptoms collected from Orphanet database (Orphanet Scientific Knowledge Files and Rare Diseases) while HSDN-MechDB uses the disease symptoms from the Human Symptoms Disease Network (HSDN) database^58^. The details are described as follows.

##### oMat-MechDB dataset

First, we collect the rare diseases and their symptom information from the Orphanet database (Version 2023) to create a symptom-disease matrix (*M*_*sd*_). Next, we keep the diseases (*D*) that belong to both the drug-disease and symptom-disease sets. Finally, we retain drugs (*R*) that each has at least one association with the diseases in *D*. After filtering, the final association matrix comprise 89 diseases and 150 drugs, with 271 associations, 13079 non-associations, and the sparsity of 97.97%. The drug-drug similarity matrix *M*_*rr*_ is calculated based on the SMILES structure using the Taminoto method. Finally, we compute the disease-disease similarity matrix *M*_*dd*_ based on the symptom-disease matrix (*M*_*sd*_) using the Gaussian interaction profile (GIP) kernel approach^10^. The details GIP method are provided in the Supplementary document.

##### HSDN-MechDB dataset

For this dataset, we follow a similar approach as performed for the oMat-MechDB dataset, but construct a symptom-disease matrix (*M*_*sd*_) using the Human Symptoms Disease Network (HSDN) database^58^, producing a large DR dataset. The HSDN-MechDB dataset comprises 616 diseases and 1270 drugs with 3710 associations and 778,619 non-associations, resulting in a high sparsity of 99.52%, see Table 1.

### Performance evaluation

#### Cross-validation

Due to the extremely high sparsity of drug-disease association matrix, we apply the stratified k-fold cross-validation (CV) approach for evaluation. Particularly, the data points are stratified into groups “1” and “0” of drug-disease pairs, then k-fold CV is applied to each group to randomly distribute data of each group into k folds. Thus, this guarantees the similar group proportion between folds. Each fold is in turn assigned as the test set, while the remaining data are used to train the prediction model. Of note, when a fold is assigned as a test set, the values of their data points in the adjacency matrix and association matrix belonging to group “1” will be set to be zero, before input into the prediction model. The aggregated predictions from all folds are then used for evaluation.

In this study, we select k = 10 for DR method evaluation. Furthermore, CV is performed *N* = 25 times, and the results are collected to evaluate the variability of the method’s predictive performance.

Of note, in each CV iteration, the adjacency matrix (see Section 4.1) is re-computed using only the training set. This updated adjacency matrix then serves as the input matrix for the prediction model, ensuring that no true associations in the test data influence the training process.

#### Evaluation metrics

After CV, we evaluate the prediction model by comparing the predicted matrix (*P*_*rd*_) with the true association matrix *M*_*rd*_. Our analysis prioritizes the top-ranked predictions for each disease, where drugs with the highest association scores are emphasized in the prediction score matrix. Specifically, we construct a ranked drug-disease association matrix (*R*_*rd*_) as follows. For each disease *d*, we sort the predicted scores *P*_.,*d*_ across all drugs in descending order, and reorder the corresponding *M*_.,*d*_ vector accordingly. The reordered *M*_.,*d*_ is then assigned to *R*_.,*d*_ to form the ranked association matrix. Now, a good prediction model will have most of the “1” values (indicating true associations) on the top of the ranked matrix *R*_*rd*_, demonstrating its ability to correctly prioritize relevant drug-disease associations.

The area under the receiver operating characteristic (ROC) curve (ROC-AUC or AUC) and area under the precision-recall curve (AUPR) are calculated based on the ranked association matrix *R*_*rd*_. AUC measures overall model performance by balancing the true positive rate (TPR) and false positive rate (FPR), making it suitable for evaluating both positive and negative predictions. For AUPR, it focuses specifically on positive associations by emphasizing precision and recall, making it particularly effective for imbalanced datasets like drug-disease associations, where positive cases are relatively rare.

### Parameter setting

The parameters for BNNR, OMC, HGIMC, VDA-GKSBMF, and DRDM are chosen based on the original implementations and recommendations provided in their papers, where they demonstrate optimal performance across most of the evaluated datasets. Specifically, the two key hyperparameters of BNNR are set to *α* = 1 and *β* = 10. Similarly, two hyperparameters of the OMC are *α* = 1 and *β* = 10. For HGIMC, the primary hyperparameters are *α* = 10 and *β* = 10, additionally, the threshold is empirically set to 0.1, and the parameter *γ* is fixed at 0.1. In the VDA-GKSBMF algorithm, three parameters $({\gamma }^{{\prime} },\omega ,{\lambda }_{1})$ need to be determined. Based on the original paper^32^, the optimal values are identified using five-fold CV, such as ${\gamma }^{{\prime} }=0.5$, *ω* = 0.4 and *λ*_1_ = 1, which provide the highest performance for three of five of their datasets^32^. Therefore, we adopt these values in the implementation of the VDA-GKSBMF model in this study. Finally, for DRDM, we follow the original implementation and set the learning rate to 0.01 with a batch size of 5120. The hidden dimension is fixed at 64, the number of graph neural iterations at 2, and the filter threshold *K* = 4. The balance terms is chosen as *ω*_1_ = *ω*_2_ = 0.6, *α*_1_ = *α*_2_ = 0.05, *β* = 0.3.

## Supplementary information

Supplementary Information

## Data Availability

All relevant datasets used in this study are available on GitHub at https://github.com/thng4165/Drug-Repurposing.

## Code availability

All relevant code used in this study are available on GitHub at https://github.com/thng4165/Drug-Repurposing.
