# Supplementary material for: Benchmarking heterogeneous network-based methods for drug repurposing
Source: NPJ Syst Biol Appl. 2025 Dec 10;12:10. doi: 10.1038/s41540-025-00633-8 (PMC12804776; doi:10.1038/s41540-025-00633-8)
Supplement: Supplementary file 1 — Supplementary Information [file 41540_2025_633_MOESM1_ESM.pdf]

Supplementary documents

1 Tanimoto coefficient

To construct the drug-drug similarity matrix, we calculate the Tanimoto coefficient between the chemical structures of drug pairs. The Tanimoto coefficient is defined as:

$$T(a,b) = \frac{|a \cap b|}{|a| + |b| - |a \cap b|} \tag{1}$$

where a and b are the sets of features present in the SMILES string of two drugs. $|a \cap b|$ is the number of common characteristics between the two drugs, while $|a|$ and $|b|$ are the total number of characteristics in each drug. The calculation was performed using *cheminformatics* package R.

2 Gaussian interaction profile (GIP) kernel

GIP kernel is determined as a function of the Gaussian distance between the interaction profiles of two diseases. The GIP kernel function [1], utilized to calculate the similarity between two diseases d_i and d_j, is defined as,

$$K\left(d_i, d_j\right) = \exp\left(-\gamma ||X_i - X_j||^2\right). \tag{2}$$

Here, $K\left(d_i, d_j\right)$ denotes the similarity between disease d_i and d_j, γ is a hyperparameter controlling the width of the Gaussian kernel, X_i and X_j represent the interaction profiles of diseases d_i and d_j respectively. A small γ results in a more localized similarity measure, emphasizing closer interactions, while a large γ yields a smoother similarity function. Following [1], γ is defined as:

$$\gamma = \frac{1}{\frac{1}{m}\sum_{i=1}^{m} |X_i|^2}. \tag{3}$$

That is, we normalize the parameter by dividing it by the average number of interactions per disease. The GIP kernel is applied to all pairs of diseases in the dataset generating a symmetric similarity matrix M_{dd} where each element $(M_{dd})_{ij}$ represents the similarity between diseases d_i and d_j.

3 Non-negative matrix factorization

Input: Given matrix V, rank $= r$, initial matrices W_0 and H_0 with $\dim(W_0) = n \times r$ and $\dim(H_0) = r \times m$, initial iteration $t = 1$
Output: $W, \quad H$

1. Initialize $W^0 = W_0$ and $H^0 = H_0$,
2. **while** not converged **do**
3. Fix W^{t-1} and update H^t by $H_{ij} \leftarrow H_{ij} \frac{\left(W^T V\right)_{ij}}{\left(W^T W H\right)_{ij}}$
4. Fix H^t and update W^t by $W_{ki} \leftarrow W_{ki} \frac{\left(V H^T\right)_{ki}}{\left(W H H^T\right)_{ki}}$
5. Test for convergence based on the change in $||V - WH||^2$
6. **if** Convergence conditions are satisfied **then**
7. Set $H = H^t$ and $W = W^t$
8. **else increment** $t = t + 1$ until convergence conditions are met
9. **end while**

4 Permutation-based NMF approach for DR: NMF-PDR

Input: Matrix V, number of permutations N
Output: Observed sample Obs, Null distributions R_0, C_0, B_0

1. For each permutation (repeat N times):

   (a) **Generate Observed Sample (Obs):**

   - Permute the positions of $V > 0$ corresponding to known associations $M_{rd} = 1$, without altering other elements of V.
   - Apply the NMF algorithm on each permuted matrix.
   - Store the NMF prediction from each permuted matrix as a column in the observed sample matrix Obs.

   (b) **Generate Null Distributions (R_0, C_0, B_0):**

   - **For R_0 (row permutation):** Permute the rows of matrix V by sampling without replacement.
   - **For C_0 (column permutation):** Permute the columns of matrix V by sampling without replacement.

- **For B_0 (row and column permutation):** Permute both rows and columns of matrix V by sampling without replacement.
- Apply the NMF algorithm on each permuted matrix (for all R_0, C_0, and B_0).
- Store the NMF predictions for each permuted matrix as a column in the corresponding null distribution matrix: R_0, C_0, or B_0.

2. Hypothesis testing and predicted values of NMF-PDR:

   - Perform three hypothesis tests to compare the predicted values of Obs with those of the null distribution R_0, C_0, and B_0.
   - Obtain predicted values of NMF-PDR by using Stouffer's Z-score method to sum the test statistics values across all tests.

5 Details of Datasets

- HDVD [2] includes 34 viruses, 219 drugs, and 455 confirmed human drug–virus associations. Drug similarity is measured based on chemical structures, while virus similarity is calculated using genomic sequences.

- LAGCN [3] includes 269 drugs, 598 diseases, and 18,416 drug–disease associations sourced from the CTD database. Drug features are collected from DrugBank, and disease terms are standardized using MeSH. Drugs are represented as binary feature vectors, enabling multiple similarity measures based on biological and chemical properties. Disease similarity is computed using MeSH-based directed acyclic graphs (DAGs).

- Fdataset [4] contains 593 drugs from DrugBank, 313 diseases from the OMIM database, and 1,933 drug–disease associations. Drug–drug similarity is measured based on chemical similarity, registered and predicted side effects, and drug target similarities, including sequence similarity, protein–protein interaction (PPI) network distance, and gene ontology (GO) semantic similarity. Disease similarity is based on the semantic similarity of disease phenotypes and the human phenotype ontology.

- Cdataset [5] includes 663 drugs from DrugBank, 409 diseases from the OMIM database, and 2,352 known drug–disease associations. Drug similarity is measured based on chemical structures, while disease similarity is assessed using phenotypes via MimMiner.

- LRSSL [6] includes 763 drugs, 681 diseases, and 3051 drug–disease associations. The chemical fingerprints defined in the PubChem database were extracted to represent the chemical substructures of drugs. Protein domains of target proteins

were obtained from InterPro database. Gene ontology information (molecular function and biological process) of target proteins was obtained from UniProt database. Then, each target protein was represented by its protein domains and gene ontology terms. The side effect information of drugs was extracted from SIDER database.

- Ydataset [7] includes 1478 drugs, 655 diseases and 8448 validated drug–disease associations. Drug similarity is measured based on chemical structures, anatomical therapeutic chemical (ATC) codes, side effects, drug–drug interactions, and target profiles. Disease similarity is assessed using phenotype similarity and ontology-based similarity.

6 Supplementary tables

Table S1: Median AUC Values: Median AUC values calculated across multiple runs for each method and dataset. The best performance for each dataset is highlighted in **bold**. The values are rounded up to the closest forth decimal place.

Dataset	NMF	NMF-PDR	NMF-DR	VDA-GKSBMF	BNNR	OMC	HGIMC	IBCF	LIBMF	DRDM
HDVD	0.7009	0.7598	0.6363	0.7779	0.7845	**0.8022**	0.7462	0.7288	0.7652	0.7431
LAGCN	0.6880	0.7076	0.6434	0.7452	**0.7943**	0.7942	0.7852	0.7993	0.7853	0.8067
Fdataset	0.8231	0.8863	0.8048	0.8706	0.9037	**0.9186**	0.8874	0.8060	0.6923	0.9279
Cdataset	0.8549	0.9090	0.8246	0.8986	0.9230	**0.9340**	0.9135	0.8393	0.7420	0.9398
LRSSL	0.7890	0.8753	0.7710	0.8724	0.8977	**0.9242**	0.8988	0.8230	0.7160	0.9355
Ydataset	0.8508	0.9115	0.8390	0.9076	**0.9171**	0.9144	0.9092	0.8547	0.7759	0.9227
oMat-MechDB	0.6787	**0.8927**	0.6678	0.8640	0.7289	0.8015	0.5927	0.6398	0.6387	0.8112
HSDN-MechDB	0.8630	**0.9291**	0.7992	0.8186	0.8307	0.8628	0.7652	0.7548	0.7299	0.9047

Table S2: Median AUPR Values: Median AUPR values calculated across multiple runs for each method and dataset. The best performance for each dataset is highlighted in **bold**. The values are rounded up to the closest forth decimal place.

Dataset	NMF	NMF-PDR	NMF-DR	VDA-GKSBMF	BNNR	OMC	HGIMC	IBCF	LIBMF	DRDM
HDVD	0.1953	0.1942	0.1037	0.2588	0.2627	**0.2777**	0.2399	0.2108	0.2298	0.2101
LAGCN	0.2326	0.2384	0.2216	0.2832	0.3439	0.3430	0.3327	**0.3591**	0.3115	0.3675
Fdataset	0.1137	0.1632	0.1238	0.1920	0.2051	**0.2149**	0.1750	0.1144	0.0335	0.2093
Cdataset	0.1281	0.1749	0.1159	0.1933	0.2257	**0.2311**	0.1969	0.1336	0.0402	0.2208
LRSSL	0.0461	0.0866	0.0422	0.1033	0.1283	**0.1350**	0.1139	0.0779	0.0186	0.1164
Ydataset	0.1276	0.1706	0.1139	0.1715	**0.2239**	0.2171	0.1998	0.1454	0.0510	0.1902
oMat-MechDB	0.0711	**0.1591**	0.0554	0.1505	0.1126	0.1254	0.0741	0.0706	0.0652	0.0919
HSDN-MechDB	0.1053	0.1315	0.0881	0.0881	0.1373	**0.1425**	0.1353	0.0724	0.0326	0.1302

Table S3: Standard deviation of overall AUC and AUPR for different methods across all datasets.

Methods	OMC	BNNR	DRDM	HGIMC	VDA-GKSBMF	NMF-PDR	IBCF	NMF	NMF-DR	LIBMF
SD of AUC	0.0614	0.0730	0.0755	0.1123	0.0582	0.0802	0.0706	0.0797	0.0848	0.0486
SD of AUPR	0.0757	0.0778	0.0860	0.0802	0.0680	0.0442	0.0974	0.0610	0.0543	0.1098

Table S4: Standard deviation of overall AUC and AUPR for different datasets across all DR methods.

Datasets	LAGCN	HDVD	Cdata	Ydata	Fdata	HSDN-MechDB	oMat-MechDB	LRSSL
SD AUC	0.0568	0.0478	0.0624	0.0482	0.0717	0.0653	0.1043	0.0725
SD AUPR	0.0555	0.0496	0.0609	0.0531	0.0569	0.0359	0.0371	0.0399

Table S5: Computational time running for each method on each dataset. The values represent the runtime (in seconds) for each method applied to a specific dataset.

Dataset	OMC	BNNR	HGIMC	VDA-GKSBMF	NMFPer	IBCF	NMF	NMF-DR	LIBMF	DRDM
HDVD	13.8338	13.8338	1.4042	2.5411	46.7428	0.9293	0.2997	28.4937	1.8541	86.71
oMat-MechDB	6.1418	6.6118	3.2966	5.7707	173.3571	1.0260	0.6233	30.6177	1.7756	135.73
LAGCN	345.5997	607.8382	67.3544	135.5319	3276.9770	21.5366	18.1400	40.8801	19.3956	10526.40
Fdata	163.5781	258.7319	63.8153	175.0659	3196.1330	25.4397	11.8188	44.5100	20.0024	3417.65
Cdata	281.3704	440.7203	130.9555	653.8371	4727.5520	43.3342	19.5164	71.5145	28.3293	6084.59
LRSSL	698.9879	1049.5000	397.6263	1077.6000	8385.0260	117.3583	39.0218	184.6867	52.8155	15871.07
hsdn-MechDB	898.7627	1214.9000	680.1562	1330.0000	39318.7300	272.3161	131.3858	443.5567	94.1662	33486.79
Ydata	1850.8000	3490.3000	945.3768	5989.9000	20124.3200	524.5496	116.1374	636.1297	119.1164	54425.34

Table S6: Performance comparison of different statistical tests for NMF-PDR method for each data. Note that NMF-PDR does not report test statistics for LAGCN when using t-test due to the small variance

Metric	Tests	HDVD	LAGCN	Fdata	Cdata	LRSSL	Ydata	oMat-MechDB	HSDN-MechDB
	t.test	0.719	NA	0.907	0.923	0.896	0.904	0.894	0.924
AUC	Ks.test	0.671	0.393	0.863	0.868	0.837	0.777	0.913	0.922
	Wilcox.test	0.762	0.707	0.888	0.909	0.876	0.912	0.885	0.929
	t-test	0.162	NA	0.162	0.166	0.085	0.162	0.120	0.093
AUPR	KS-test	0.106	0.093	0.136	0.121	0.034	0.034	0.197	0.141
	Wilcox-test	0.214	0.238	0.168	0.174	0.083	0.171	0.159	0.134

7 Supplementary figures

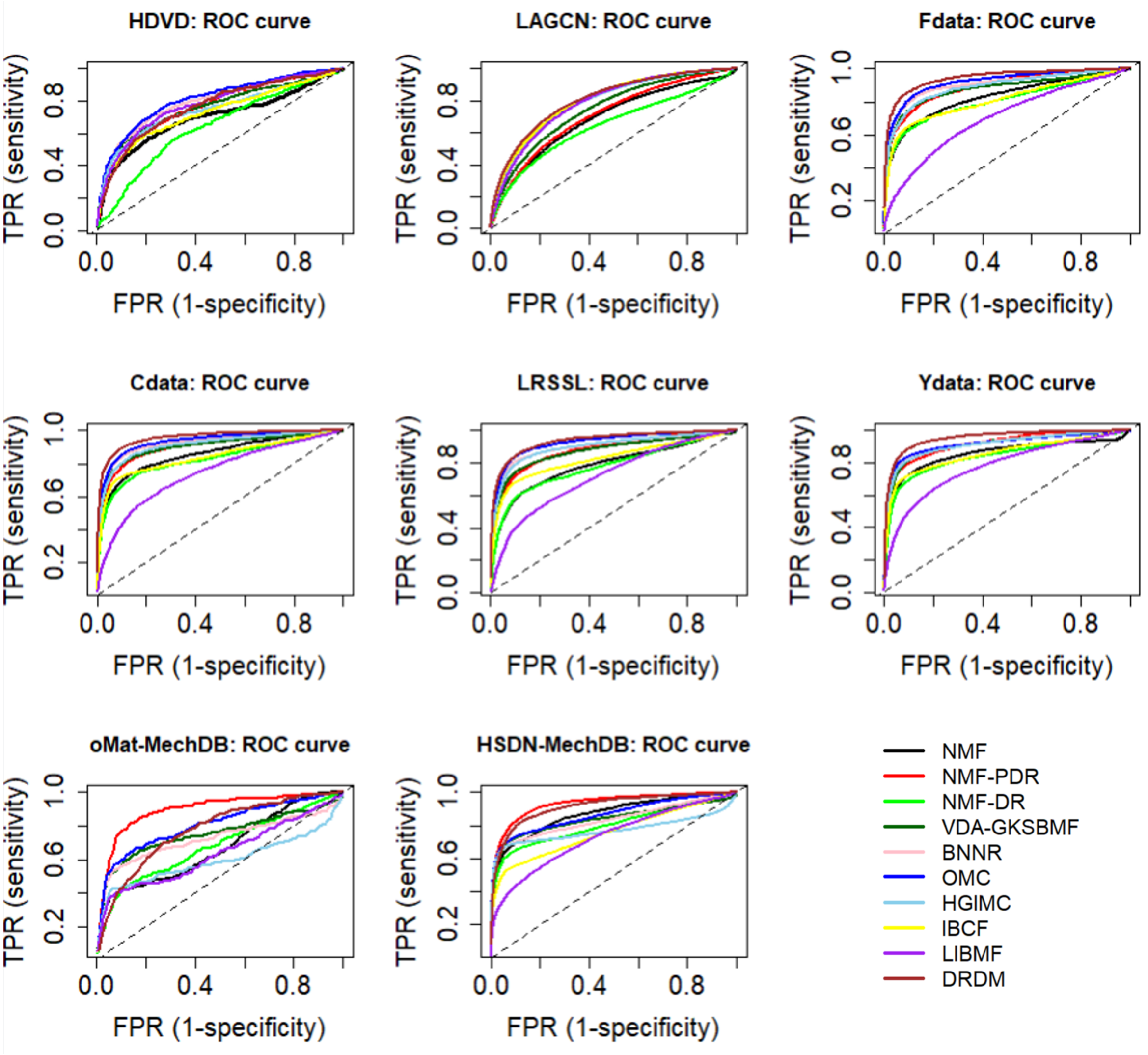

Figure S1: The performance of all methods in predicting drug–disease association for 10-fold cross-validation for each dataset: ROC curve of prediction results.

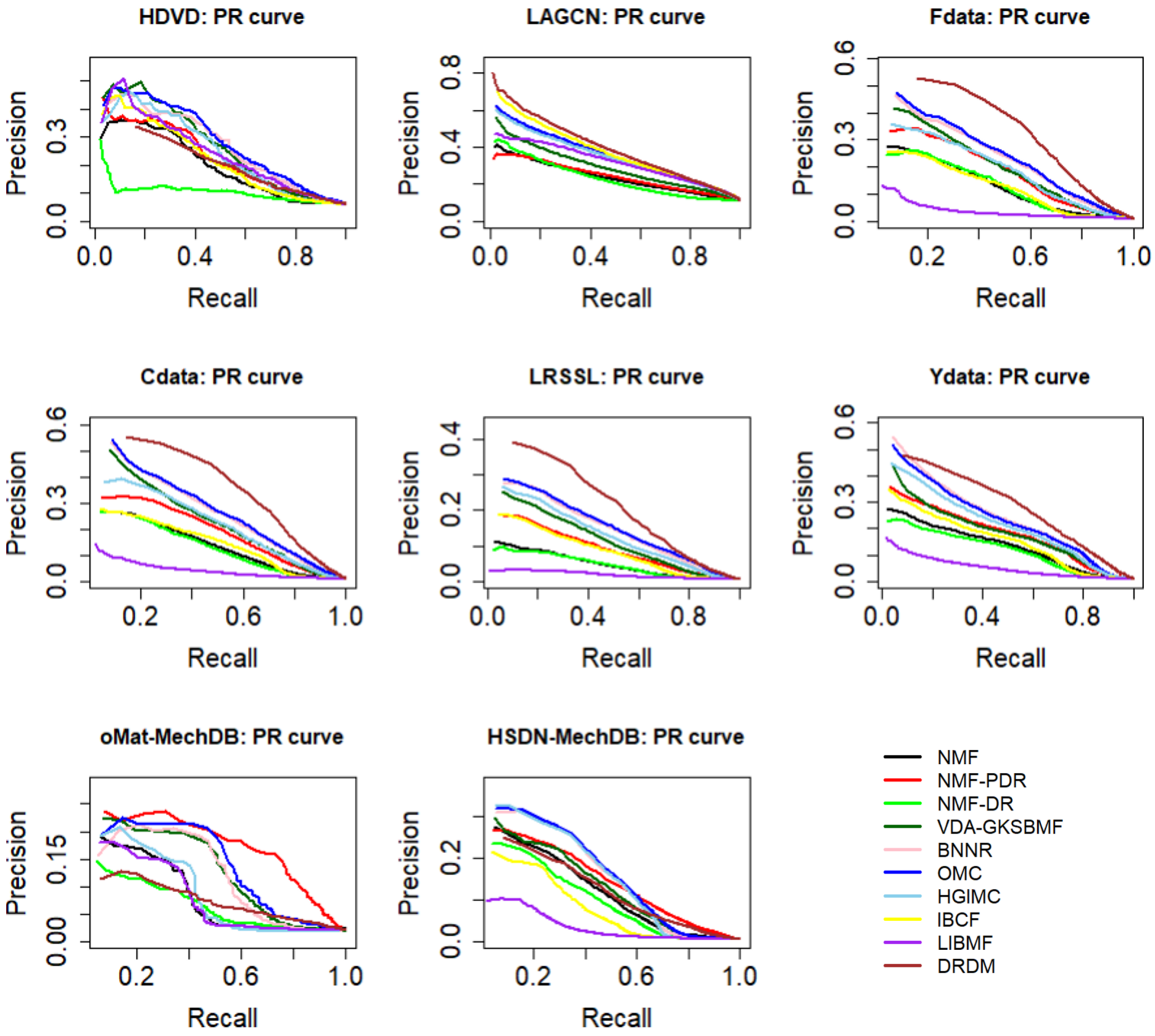

Figure S2: The performance of all methods in predicting drug–disease association for 10-fold cross-validation for each dataset: Precision-Recall (PR) curve of predicting candidate diseases for drugs.

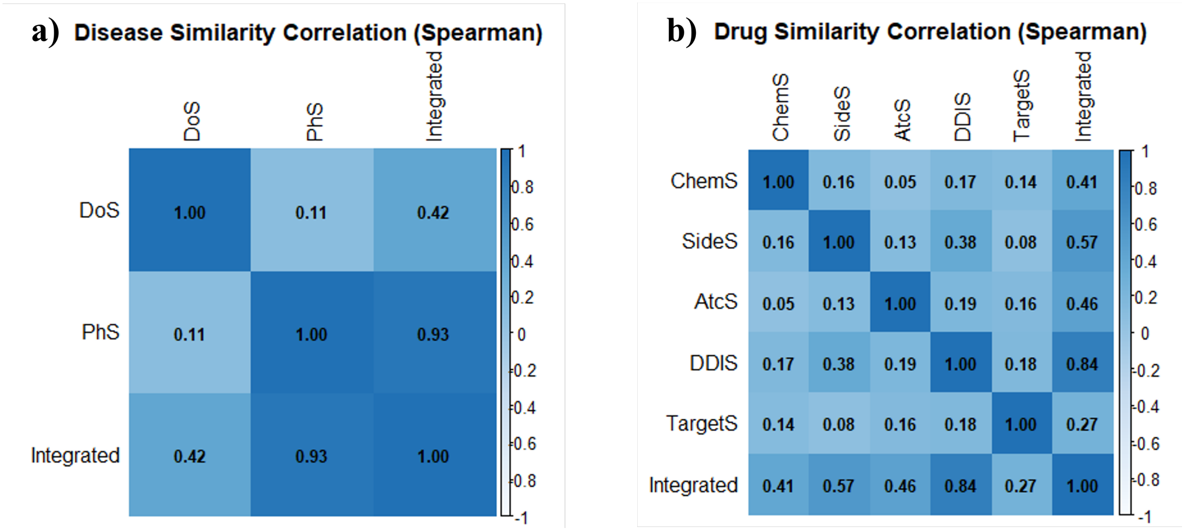

Figure S3: Spearman correlation heatmaps of similarity matrices for the Fdataset.

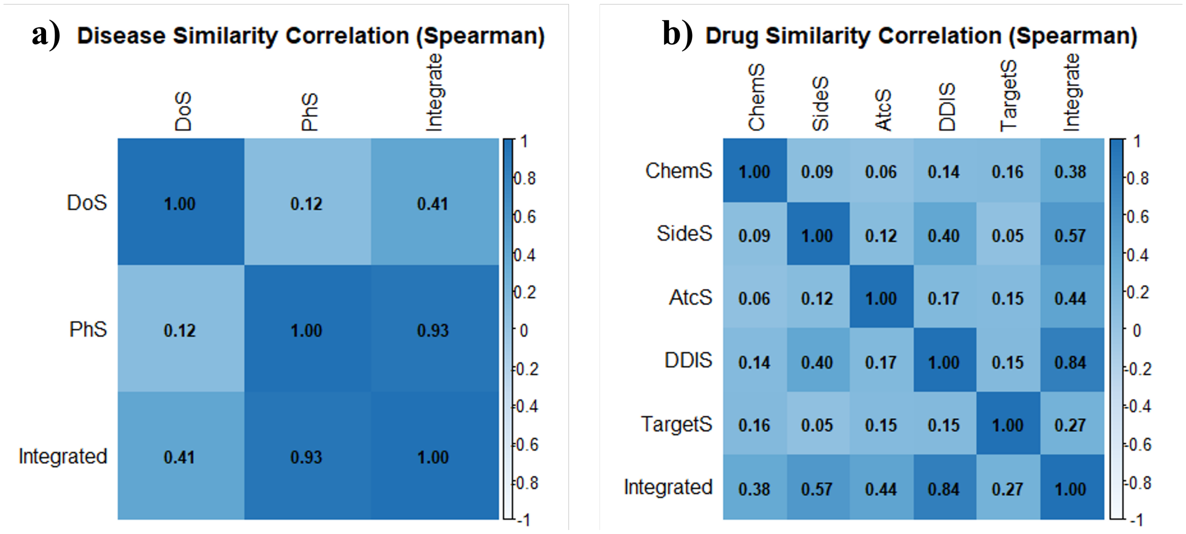

Figure S4: Spearman correlation heatmaps of similarity matrices for the Cdataset.

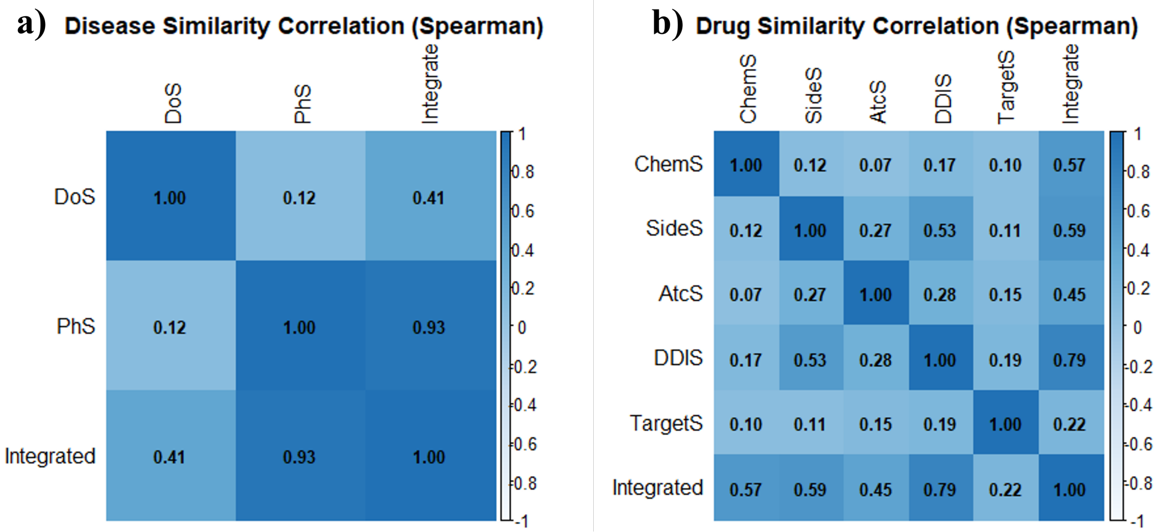

Figure S5: Spearman correlation heatmaps of similarity matrices for the Ydataset.

References

[1] Twan Van Laarhoven, Sander B Nabuurs, and Elena Marchiori. Gaussian interaction profile kernels for predicting drug–target interaction. *Bioinformatics*, 27(21):3036–3043, 2011.

[2] Yajie Meng, Min Jin, Xianfang Tang, and Junlin Xu. Drug repositioning based on similarity constrained probabilistic matrix factorization: Covid-19 as a case study. *Applied soft computing*, 103:107135, 2021.

[3] Wen Zhang, Xiang Yue, Weiran Lin, Wenjian Wu, Ruoqi Liu, Feng Huang, and Feng Liu. Predicting drug-disease associations by using similarity constrained matrix factorization. *BMC bioinformatics*, 19:1–12, 2018.

[4] Assaf Gottlieb, Gideon Y Stein, Eytan Ruppin, and Roded Sharan. Predict: a method for inferring novel drug indications with application to personalized medicine. *Molecular systems biology*, 7(1):496, 2011.

[5] Huimin Luo, Jianxin Wang, Min Li, Junwei Luo, Xiaoqing Peng, Fang-Xiang Wu, and Yi Pan. Drug repositioning based on comprehensive similarity measures and bi-random walk algorithm. *Bioinformatics*, 32(17):2664–2671, 2016.

[6] Xujun Liang, Pengfei Zhang, Lu Yan, Ying Fu, Fang Peng, Lingzhi Qu, Meiying Shao, Yongheng Chen, and Zhuchu Chen. Lrssl: predict and interpret drug–disease associations based on data integration using sparse subspace learning. *Bioinformatics*, 33(8):1187–1196, 2017.

[7] Mengyun Yang, Gaoyan Wu, Qichang Zhao, Yaohang Li, and Jianxin Wang. Computational drug repositioning based on multi-similarities bilinear matrix factorization. *Briefings in bioinformatics*, 22(4):bbaa267, 2021.
